# Prediction of post-treatment hypothyroidism using changes in thyroid volume after radioactive iodine therapy in adolescent patients with Graves' disease

**DOI:** 10.1186/1687-9856-2011-14

**Published:** 2011-11-07

**Authors:** Nobuhiro Nakatake, Shuji Fukata, Junichi Tajiri

**Affiliations:** 1 Tajiri Thyroid Clinic, Kumamoto 862-0950, Japan

## Abstract

**Background:**

The goal of iodine-131 therapy for pediatric Graves' disease is to induce hypothyroidism. However, changes in post-treatment thyroid volume have not been investigated in pediatric and/or adolescent patients.

**Objective:**

The aim of this retrospective study was to examine whether changes in thyroid volume predict post-treatment hypothyroidism in adolescent Graves' disease patients.

**Patients and Methods:**

We used ultrasonography to examine changes in thyroid volume, and also assessed thyroid functions, at 0, 1, 3, 5, 8 and 12 months after iodine-131 treatment in 49 adolescents ranging in age from 12 to 19 years retrospectively. Based on thyroid function outcome at 12 months, patients were divided into two groups: 29 patients with overt hypothyroidism requiring levothyroxine replacement and 20 without overt hypothyroidism. We compared changes in post-radioiodine thyroid volume between the two groups.

**Results:**

About 90% of patients whose thyroid volume at 3 months after iodine-131 administration was less than 50% of the original volume were hypothyroid by one year after treatment (positive predictive value 88%, sensitivity 75.9%, specificity 85.0%).

**Conclusions:**

We believe ultrasonographic measurement of thyroid volume at 3 months after iodine-131 to be clinically useful for predicting post-treatment hypothyroidism in adolescent Graves' disease patients.

## Introduction

Graves' disease (GD) is the most common cause of hyperthyroidism in children, adolescents and adults [1-3]. Treatments available for GD include anti-thyroid medications (methimazole or propylthiouracil), surgery and radioactive iodine (RAI) [4,5]. There is ongoing debate worldwide regarding the most suitable therapy for GD in pediatric patients. Although anti-thyroid medications are commonly used as first-line therapy for pediatric GD, long-term remission occurs in only 20% to 30% of pubertal cases and 15% of pre-pubertal cases treated pharmacologically [3,6-8]. Consequently, either surgery or RAI is needed to achieve a long-term cure in most pediatric GD patients.

RAI therapy is generally considered to be safe, inexpensive and effective, with relatively few side effects [8-10]. Radioiodine was introduced for the treatment of GD more than 50 years ago [11], and at present is the most commonly used treatment for adult GD in the North America [12]. In 107 young GD patients who had been treated with RAI before age 20 years, no increased risk of adverse events was reported [13]. In some facilities, RAI is becoming the first-line therapy for GD in children and adolescents [14,15].

The goal of iodine-131 therapy for pediatric GD is to induce hypothyroidism [16,17]. When children are treated with 330 μCi/g of iodine-131, hypothyroidism is achieved in nearly 95% of patients [18]. Higher dose ablative therapy (13.8 to 15.6 mCi) is effective in nearly all children with GD [19]. The use of high dose iodine-131 will destroy most thyroid tissue, thereby decreasing the risk of RAI-induced thyroid tumors, and is thus preferable especially in children [20]. The long-term risks of thyroid cancer appear to be lower when the thyroid gland is largely ablated than when residual thyroid tissue remains [21,22]. Changes in post-RAI thyroid volume have been investigated in adult GD patients [10,23-26], but not in pediatric and/or adolescent patients [3].

The objective of this retrospective study was to investigate changes in post-radioiodine thyroid volume in adolescent GD patients (< 20 years old) and also to examine whether these changes predict post-treatment hypothyroidism.

## Patients and Methods

The medical records of all adolescent patients (< 20 years old) at Tajiri Thyroid Clinic who received a single RAI treatment for GD during the decade from January 2000 to January 2010 were examined retrospectively. The present study was approved by the Institutional Review Board of our clinic.

GD was diagnosed based on elevated free thyroxine and suppressed thyrotropin concentrations, elevated TSH receptor antibodies (TRAb), and diffuse, elevated uptake of radioiodine or technetium-99 m within the thyroid. Thyrotropin, free thyroxine and TSH receptor antibody were measured by electrochemiluminescence immunoassay (Cobas e601; Roche Diagnostics, Tokyo, Japan).

The iodine-131-absorbed radiation dose was calculated from RAIU and thyroid weight, using the formula: dose (μCi/g) = oral iodine-131 dose (mCi) × estimated 24 h RAIU (%) × 10/thyroid weight (g). Twenty-four hour RAI uptake was estimated using 4-hour uptake of iodine-123 [27] or 20-minute uptake of technetium-99 m [28]. Thyroid volume was estimated by ultrasound (SSA-350A; Toshiba Inc. Ltd., Tokyo, Japan) as previously reported [29]. Thyroid function (free thyroxine and thyrotropin) and ultrasonographic thyroid volume were determined at 1, 3, 5, 8 and 12 months after RAI therapy. When free thyroxine values dropped below 0.8 ng/dL and/or thyrotropin levels rose above 20 μIU/mL, replacement therapy with levothyroxine was initiated.

Statistical analyses were performed using Student's t test and the chi-squared test. Values are shown as means ± standard deviation (SD). A P-value of less than 0.05 was considered to indicate a statistically significant difference.

## Results

There were 10 males and 39 females ranging in age from 12 to 19 years (mean ± SD, 16.4 ± 1.8 years old). All 49 patients were initially treated with anti-thyroid medications for 1 to 108 months (80% with methimazole, 20% with propylthiouracil). RAI therapy was performed due to lack of remission after 14 to 108 months (39%) of medical treatment, the development of a presumed toxic reaction to anti-thyroid drugs (44%; rash, arthralgia, hepatitis, neutropenia), or a desire for definitive therapy (17%). Anti-thyroid drugs were discontinued 3 to 5 days before RAI treatment. After administration of iodine-131, patients were treated with anti-thyroid drugs with or without propranolol to control symptoms of hyperthyroidism until hyperthyroxinemia abated.

The mean RAI dose for our 49 patients was 184 ± 84 μCi/g (range 44 - 393 μCi/g) and mean thyroid volume decreased significantly from 34.5 ml to 8.3 ml during the one year period after RAI (P < 0.00001). Based on thyroid functions at one year after RAI, patients were divided into two groups: 29 (59.2%) with overt hypothyroidism (hypothyroid patients) requiring levothyroxine replacement therapy (mean time until hypothyroidism: 4 ± 1.5 months, range 1 - 8 months) and 20 (40.8%) without hypothyroidism (non-hypothyroid patients) taking no medication at one year after RAI. The 20 non-hypothyroid patients consisted of 8 euthyroid and 12 hyperthyroid patients. Euthyroid patients included two who experienced transient hypothyroidism at 3 months after I-131 but had recovered without intervention at 1 year and one with subclinical hypothyroidism (TSH 6.53 μIU/ml (normal range: 0.20 - 3.30 μIU/ml)) at 1 year. Hyperthyroidism was subclinical in 8 patients and mild in 4 with serum free T4 levels of 1.93 - 2.03 ng/dl (normal range: 0.90 - 1.80 ng/dl). None of the hyperthyroid patients took anti-thyroid drugs at one year after RAI because all were asymptomatic. There were no statistically significant differences between euthyroid and hyperthyroid patients with respect to pre- and post-treatment thyroid volumes, or in percent volume reductions at 1, 3, 5, 8 and 12 months. The characteristics of adolescent GD patients who received RAI therapy, divided into hypothyroid and non-hypothyroid groups, are summarized in Table 1. There were no significant differences between the two groups in gender (*P *= 0.20), age (*P *= 0.18), pre-treatment thyroid volume (*P *= 0.30) or pre-treatment TRAb values measured by cosmic TRAb coated-tube kit (*P *= 0.45). The two groups differed only in the RAI dose administered (*P *= 0.048).

**Table 1:** Characteristics of Adolescent GD Patients Receiving Iodine-131 Therapy

	Hypothyroid (n = 29)	Non-hypothyroid (n = 20)	P-value
Gender, female/male	20/9	19/1	NS (0.06)
Age (yr)	16.1 ± 1.9	16.8 ± 1.6	NS (0.18)
Pre-treatment thyroid volume (ml)	36.3 ± 17.5	31.9 ± 11.6	NS (0.30)
Pre-treatment TRAb (%)	60.2 ± 27.8 (n = 22)	53.5 ± 29.1	NS (0.45)
I-131 dose (μCi/g)	202.7 ± 88.6	156.3 ± 71.2	0.048

NS: not significant. All values are means ± SD.

**Figure 1:**
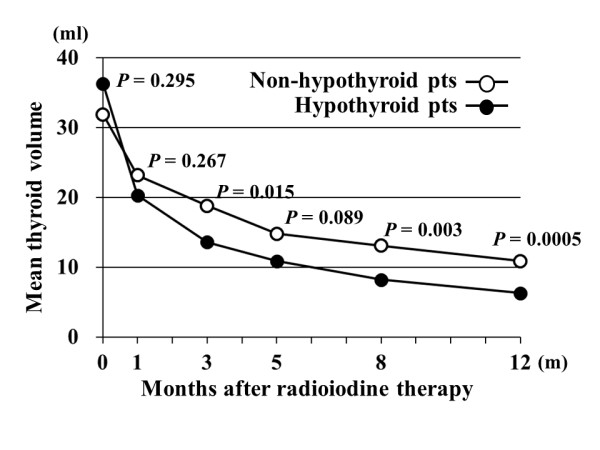
**Changes in thyroid volume during the one year period after iodine-131 therapy in hypothyroid and non-hypothyroid patients**. Mean thyroid volumes in hypothyroid vs. non-hypothyroid patients were 36.3 ml vs. 31.9 ml at 0 months (m), 20.3 ml vs. 23.2 ml at 1 m, 13.6 ml vs. 18.8 ml at 3 m, 10.9 ml vs. 14.8 ml at 5 m, 8.2 ml vs. 13.1 ml at 8 m, and 6.3 ml vs. 10.9 ml at 12 m after iodine-131 therapy.

**Figure 2:**
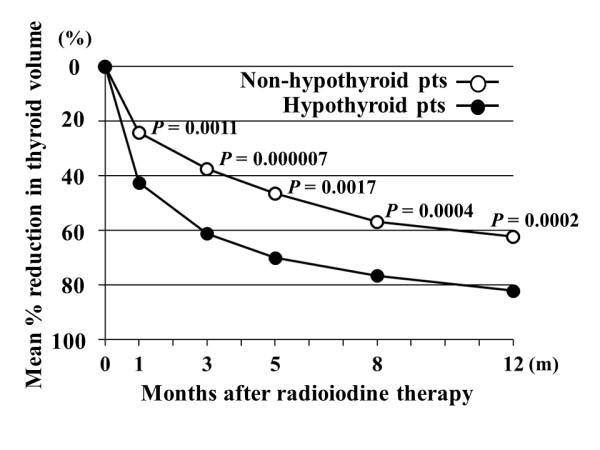
**Percent reductions in thyroid volume during the one year period after iodine-131 therapy in hypothyroid and non-hypothyroid patients**. Mean percent reductions in thyroid volume in hypothyroid vs. non-hypothyroid patients were 42.6% vs. 24.2% at 1 month (m), 61.2% vs. 37.6% at 3 m, 70.1% vs. 46.5% at 5 m, 76.6% vs. 56.9% at 8 m, and 82.2% vs. 62.4% at 12 m after iodine-131 therapy.

We compared changes in post-RAI administration thyroid volume during the one year period after treatment between hypothyroid and non-hypothyroid patients. As shown in Figure 1, post-treatment thyroid volume was significantly smaller in hypothyroid than in non-hypothyroid patients, especially after 8 months (8.2 ml vs. 13.1 ml at 8 months; P = 0.003, 6.3 ml vs. 10.9 ml at 12 months; P = 0.0005). The mean percent reduction in thyroid volume was significantly greater in hypothyroid than in non-hypothyroid patients at all measurement time points (P < 0.005) (Figure 2). We examined whether changes in thyroid volume predict post-treatment hypothyroidism at one year. The optimal cut-off point for predicting post-treatment hypothyroidism is considered to be a 50% reduction, as compared to the original volume of the thyroid gland, at 3 months after iodine-131 administration (Table 2). The sensitivity, specificity, positive predictive value, negative predictive value, positive likelihood ratio, and negative likelihood ratio were 75.9%, 85.0%, 88.0%, 70.8%, 5.1 and 0.3, respectively.

**Table 2:** Sensitivity and Specificity of Percent Reductions in Thyroid Volume at Each Measurement Time Point after Iodine-131 Therapy

	Month(s) after iodine-131 therapy				
	1	3	5	8	12
% reduction*	Sens Spec	Sens Spec	Sens Spec	Sens Spec	Sens Spec
30%	75.9 55.0				
40%	55.2 80.0	79.3 60.0			
50%	41.4 95.0	75.9 85.0	92.6 53.3		
60%		55.2 90.0	73.3 74.1	96.2 57.9	
70%			51.9 93.3	73.1 78.9	85.2 60.0
80%				46.2 94.7	74.1 85.0
90%					18.5 100.0

*% reduction: cut-off point for percent reduction in thyroid volume

Sens: sensitivity for predicting hypothyroidism at one year after iodine-131 therapy using %reduction

Spec: specificity for predicting hypothyroidism at one year after iodine-131 therapy using %reduction

We also examined the relationship between iodine-131 doses and post-RAI administration thyroid volume at 3 months in all 49 patients. Three iodine-131 doses (mean ± SD) were compared: 120 ± 32 μCi/g (n = 25, range 44 - 171 μCi/g), 200 ± 20 μCi/g (n = 11, range 174 - 224 μCi/g) and 300 ± 62 μCi/g (n = 13, range 225 - 393 μCi/g). When doses of 120 μCi/g, 200 μCi/g and 300 μCi/g were used, post-RAI administration thyroid volumes at 3 months were less than 50% of the pre-treatment thyroid volume in 36% (9/25), 55% (6/11) and 85% (11/13) of patients, respectively. We also found that doses of 120 μCi/g, 200 μCi/g and 300 μCi/g resulted in hypothyroidism at 1 year after RAI in 48% (12/25), 55% (6/11) and 85% (11/13) of the patients, respectively.

## Discussion

This retrospective study showed mean post-treatment thyroid volume to be significantly decreased, from 34.5 ml to 8.3 ml (P < 0.00001), at one year after RAI in 49 adolescent GD patients (age range: 12 - 19 years), as has been demonstrated in adult GD patients (10,23-26).

The goal of iodine-131 therapy for pediatric GD is to ablate the thyroid gland, in order to decrease the risk of RAI-induced thyroid tumors [18]. However, changes in post- RAI administration thyroid volume have not been investigated in pediatric and/or adolescent patients [3]. We found thyroid volume at one year after RAI administration for adolescent Graves' hyperthyroidism to be significantly smaller in hypothyroid than in non-hypothyroid patients (mean 6.3 ml vs. 10.9 ml; P < 0.001). As the post- RAI administration thyroid volume is smaller in hypothyroid patients, apparently conferring a lower risk of thyroid neoplasm development, this underscores the need for hypothyroidism to be a goal of therapy when using iodine-131 to treat GD in children [18].

A correlation between changes in thyroid volume and thyroid function outcome in adult patients with GD has been described (10,23-25). However, these studies did not investigate the relationship between the degree of thyroid volume reduction and thyroid function outcome. Chiovato et al reported that the degree of thyroid volume reduction after RAI administration was the best predictor of early (within 1 year) thyroid function outcome in adult Graves' hyperthyroidism [26]. In fact, we found that about 90% of our patients had become hypothyroid at one year when thyroid volume was less than 50% of the original volume at 3 months after iodine-131 administration (positive predictive value 88%, sensitivity 75.9%, specificity 85.0%).

We also found that 85% of patients treated with a dose of 300 μCi/g (range: 225 - 393 μCi/g) showed remarkable thyroid gland shrinkage (< 50% of the original thyroid gland volume at 3 months) and 85% were hypothyroid at one year. These data indicate that doses of approximately 300 μCi/g are needed to insure ablation of thyroid tissue. Our findings are thus consistent with those reported by Rivkees et al [18].

High thyroid-stimulating antibody levels before iodine-131 seem to be associated with a relative resistance to therapy (24, 26). On the other hand, TRAb levels did not show any predictive value for iodine-131 therapeutic outcome [25]. In our present study, pre-treatment TRAb values were not correlated with iodine-131 therapeutic outcome.

In conclusion, thyroid volume progressively diminished for one year after iodine-131 administration for adolescent GD. Decreases were more significant in hypothyroid than in non-hypothyroid patients. We also demonstrated that approximately 90% of patients became hypothyroid within one year when thyroid volume was less than 50% of the original volume at 3 months after RAI therapy. We believe ultrasonographic thyroid volume measurement at 3 months after iodine-131 administration to be clinically useful for predicting post-treatment hypothyroidism.

## Competing interests

The authors declare that they have no competing interests.

## Authors' contributions

All authors contributed to the development and writing of this manuscript and each has many years of clinical experience in the care of individuals with Graves' disease. All authors read and approved the final manuscript.

## References

[B1] Saxena KM Crawford JD Talbot NB Childhood thyrotoxicosis: a longer term perspective Br Med J 1964 2 1153 1158 10.1136/bmj.2.5418.1153 14190483 PMC1816770

[B2] Brent GA Clinical practice. Graves' disease N Engl J Med 2008 358 2594 2605 10.1056/NEJMcp0801880 18550875

[B3] Rivkees SA Sklar C Freemark M Clinical review 99: the management of Graves' disease in children, with special emphasis on radioiodine treatment J Clin Endocrinol Metab 1998 83 3767 3775 10.1210/jc.83.11.3767 9814445

[B4] Franklyn JA The management of hyperthyroidism N Engl J Med 1994 330 1731 1738 10.1056/NEJM199406163302407 7910662

[B5] Levy WJ Schumacher P Gupta M Treatment of childhood Graves' disease. A review with emphasis on radioiodine treatment Cleve Clin J Med 1988 55 373 382 2457461 10.3949/ccjm.55.4.373

[B6] Hamburger JI Management of hyperthyroidism in children and adolescents J Clin Endocrinol Metab 1985 60 1019 1024 10.1210/jcem-60-5-1019 2579967

[B7] Lazar L Kalter-Leibovici O Pertzelan A Weintrob N Josefsberg Z Phillip M Thyrotoxicosis in prepubertal children compared with pubertal and postpubertal patients J Clin Endocrinol Metab 2000 85 3678 3682 10.1210/jc.85.10.3678 11061522

[B8] Shulman DI Muhar I Jorgensen EV Diamond FB Bercu BB Root AW Autoimmune hyperthyroidism in prepubertal children and adolescents: comparison of clinical and biochemical features at diagnosis and responses to medical therapy Thyroid 1997 7 755 760 10.1089/thy.1997.7.755 9349579

[B9] Spencer RP Kayani N Karimeddini MK Radioiodine therapy of hyperthyroidism: socioeconomic considerations J Nucl Med 1985 26 663 665 3998855

[B10] Nygaard B Hegedüs L Gervil M Hjalgrim H Hansen BM Søe-Jensen P Hansen JM Influence of compensated radioiodine therapy on thyroid volume and incidence of hypothyroidism in Graves' disease J Intern Med 1995 238 491 497 10.1111/j.1365-2796.1995.tb01230.x 9422034

[B11] Chapman EM History of the discovery and early use of radioactive iodine JAMA 1983 250 2042 2044 10.1001/jama.250.15.2042 6352970

[B12] Ma C Kuang A Xie J Liu GJ Radioiodine treatment for pediatric Graves' disease (Review) The Cochrane Library 2009 3 1 31 10.1002/14651858.CD006294.pub2 18646146

[B13] Read CH Jr Tansey MJ Menda Y A thirty-six year retrospective analysis of the efficacy and safety of radioactive iodine in treating young Graves' patients J Clin Endocrinol Metab 2004 89 4229 4233 10.1210/jc.2003-031223 15356012

[B14] Foley TP Jr Charron M Radioiodine treatment of juvenile Graves' disease Exp Clin Endocrinol Diabetes 1997 4 Suppl 105 61 65 10.1055/s-0029-1211936 9439919

[B15] Chao M Jiawei X Guoming W Jianbin L Wanxia L Driedger A Shuyao Z Qin Z Radioiodine treatment for pediatric hyperthyroid Grave's disease Eur J Pediatr 2009 168 1165 1169 10.1007/s00431-009-0992-2 19421775

[B16] Rivkees SA Pediatric Graves' Disease: Controversies in Management Horm Res Paediatr 2010 74 305 311 10.1159/000320028 20924158

[B17] Bahn Chair RS Burch HB Cooper DS Garber JR Greenlee MC Klein I Laurberg P McDougall IR Montori VM Rivkees SA Ross DS Sosa JA Stan MN Hyperthyroidism and Other Causes of Thyrotoxicosis: Management Guidelines of the American Thyroid Association and American Association of Clinical Endocrinologists Thyroid 2011 21 593 646 10.1089/thy.2010.0417 21510801

[B18] Rivkees SA Cornelius EA Influence of iodine-131 dose on the outcome of hyperthyroidism in children Pediatrics 2003 111 745 749 10.1542/peds.111.4.745 12671107

[B19] Nebesio TD Siddiqui AR Pescovitz OH Eugster EA Time course to hypothyroidism after fixed-dose radioablation therapy of Graves' disease in children J Pediatr 2002 141 99 103 10.1067/mpd.2002.125494 12091858

[B20] Rivkees SA Dinauer C Controversy in clinical endocrinology. An optimal treatment for pediatric Graves' disease is radioiodine J Clin Endocrinol Metab 2007 92 797 800 17341574 10.1210/jc.2006-1239

[B21] Ron E Doody MM Becker DV Brill AB Curtis RE Goldman MB Harris BS Hoffman DA McConahey WM Maxon HR Preston-Martin S Warshauer ME Wong FL Boice JD Jr Cancer mortality following treatment for adult hyperthyroidism. Cooperative Thyrotoxicosis Therapy Follow-up Study Group JAMA 1998 280 347 355 10.1001/jama.280.4.347 9686552

[B22] Dobyns BM Sheline GE Workman JB Tompkins EA McConahey WM Becker DV Malignant and benign neoplasms of the thyroid in patients treated for hyperthyroidism: a report of the Cooperative Thyrotoxicosis Therapy Follow-up Study J Clin Endocrinol Metab 1974 38 976 998 10.1210/jcem-38-6-976 4134013

[B23] Peters H Fischer C Bogner U Reiners C Schleusener H Reduction in thyroid volume after radioiodine therapy of Graves' hyperthyroidism: results of a prospective, randomized, multicentre study Eur J Clin Invest 1996 26 59 63 10.1046/j.1365-2362.1996.98243.x 8682157

[B24] Murakami Y Takamatsu J Sakane S Kuma K Ohsawa N Changes in thyroid volume in response to radioactive iodine for Graves' hyperthyroidism correlated with activity of thyroid-stimulating antibody and treatment outcome J Clin Endocrinol Metab 1996 81 3257 3260 10.1210/jc.81.9.3257 8784079

[B25] Gómez-Arnaiz N Andía E Gumà A Abós R Soler J Gómez JM Ultrasonographic thyroid volume as a reliable prognostic index of radioiodine-131 treatment outcome in Graves' disease hyperthyroidism Horm Metab Res 2003 35 492 497 12953167 10.1055/s-2003-41807

[B26] Chiovato L Fiore E Vitti P Rocchi R Rago T Dokic D Latrofa F Mammoli C Lippi F Ceccarelli C Pinchera A Outcome of thyroid function in Graves' patients treated with radioiodine: Role of thyroid-stimulating and thyrotropin-blocking antibodies and of radioiodine-induced thyroid damage J Clin Endocrinol Metab 1998 83 40 46 10.1210/jc.83.1.40 9435414

[B27] Vemulakonda US Atkins FB Zissman HA Therapy dose calculation in Graves' disease using early I-123 uptake measurements Clin Nucl Med 1996 21 102 105 10.1097/00003072-199602000-00004 8697676

[B28] Smith JJ Croft BY Brookeman VA Teates CD Estimation of 24-hour thyroid uptake of I-131 sodium iodide using a 5-minute uptake of technetium-99m pertechnetate Clin Nucl Med 1990 15 80 83 10.1097/00003072-199002000-00003 2155732

[B29] Tajiri J Radioactive iodine therapy for goitrous Hashimoto's thyroiditis J Clin Endocrinol Metab 2006 91 4497 4500 10.1210/jc.2006-1163 16895949

